# Arrhythmias in the Age of Coronavirus Disease 2019

**DOI:** 10.19102/icrm.2021.120107

**Published:** 2021-01-15

**Authors:** Imran Niazi, Mohsin Khan

**Affiliations:** ^1^Aurora Cardiovascular and Thoracic Services, Aurora Sinai/Aurora St. Luke’s Medical Centers, University of Wisconsin School of Medicine and Public Health

**Keywords:** Arrhythmias, antiarrhythmics, atrial fibrillation, COVID-19, ventricular tachycardia

The year 2020 has brought with it an unprecedented challenge to health care in the form of the coronavirus disease 2019 (COVID-19) pandemic. Caused by severe acute respiratory syndrome coronavirus 2 (SARS-CoV-2), a poorly understood but highly infectious agent, this disease can manifest with a wide spectrum of illness, involving multiple organ systems including the heart. Newly developed treatments can have unknown cardiac electrophysiologic effects, while repurposed medications may interact with heart rhythm medications to varying degrees.

Here, we seek to review the knowledge accumulated to date regarding the various tachyarrhythmias and bradyarrhythmias associated with COVID-19 infection and the specific challenges involved when treating patients infected with SARS-CoV-2.

## COVID-19 and the heart

Research from early in the pandemic suggests that cardiac injury (defined as troponin elevation above the 99^th^ percentile) was present in about 20% to 30% of hospitalized COVID-19 patients in China and was associated with a much higher mortality rate than that in those without cardiac injury.^1,2^ Even patients not sick enough to require hospitalization may not escape experiencing at least a temporary cardiac pathology: a prospective observational study using cardiac magnetic resonance data collected from 100 patients in the University Hospital Frankfurt COVID-19 Registry at a median of 71 days after recovery from COVID-19 noted abnormal findings suggestive of cardiac involvement in 78 patients (78%). Two-thirds of these patients had recovered from COVID-19 at home. Moreover, troponin-T levels were found to be elevated during high-sensitivity cardiac troponin testing in 71 patients at the time of cardiac magentic resonance imaging.^3^

## Cardiac arrhythmias

Cardiac arrhythmias occur with increased frequency in patients with COVID-19, especially in those sufficiently ill enough to require critical care. Other than sinus tachycardia, atrial fibrillation and flutter are probably the most frequent arrhythmias that the treating physician can expect to confront in this patient population. In 138 patients hospitalized in Hubei province in China, the incidence rate of atrial arrhythmias was 44% in the intensive care unit setting,^4^ while the incidence rate of the same among 393 ICU patients admitted to a tertiary care center in New York City was 17.7%.^5^ Meanwhile, ventricular tachycardia occurred in only one patient (0.3%) in the latter study.^5^ Bradyarrhythmias are less common, with anecdotal evidence suggesting the hyperadrenergic state seen in severely ill patients causes tachyarrhytmias more often than bradycardia, and, while heart block has been reported, it is rare and usually transient.^6^

The frequent occurrence of atrial fibrillation and flutter in patients hospitalized for COVID-19 may be due to a number of underlying causes, including the patient’s existence in a hyperadrenergic state as well as the onset of hypoxia, pulmonary hypertension, and viral myocarditis. Other contributing factors (which may cause ventricular arrhythmias as well) include immune-mediated injury caused by inflammatory cytokines, which can prolong the action-potential duration by interacting with potassium and calcium channels, and myocardial ischemia due to virus-mediated vasculitis, viral myocarditis, or underlying coronary atherosclerosis.^7^ Ventricular arrhythmias can be mediated by all of the above, but the “off-label” use of some agents with known arrhythmic potential is of particular concern, especially in the absence of any proven benefit. Chief among these medications are hydroxychloroquine, chloroquine, and azithromycin, all of which prolong the QT interval by interacting with the hERG potassium channel and can precipitate torsades de pointes when used individually or in combination.^8^ The protease inhibitors lopinavir and ritonavir have also been used empirically^9^; these drugs inhibit cytochrome P450 3A4 and reduce the hepatic clearance of antiarrhythmics such as amiodarone, propafenone, flecainide, and quinidine. However, their use has declined in the United States because of lack of efficacy and the recent availability of the antiviral remdesivir. Although we do not yet have extensive experience with remdesivir, a double-blind randomized National Institutes of Health trial involving 1,062 patients did not confirm any significant electrocardiogram changes or proarrhythmic effects to be associated with this drug.^10^

**Table 1** summarizes the known arrhythmic effects of COVID-19 drugs and their interactions with antiarrhythmic agents.

## Management strategies

There are insufficient published studies to guide the management of arrhythmias due to COVID-19 at this time. Some guidance may be obtained from a European Society of Cardiology consensus document.^11^ However, this was last updated in June 2020 and predates fresh data on antiviral and immune-modulating drugs as well as therapies developed since. At the time of its publication, Europe was the epicenter of the pandemic and very little was known about the disease. In the absence of proven therapies, off-label use of repurposed drugs based on the thinnest of preliminary laboratory evidence was rampant. Among the drugs used were chloroquine, hydroxychloroquine, and azithromycin, all of which are known to prolong the QT interval. Also used were anti–human immunodeficiency virus protease inhibitors, which have similar effects and interact with the hepatic metabolism of commonly used antiarrhythmic drugs. This may explain the reluctance to recommend class III antiarrhythmics in the European Society of Cardiology guidance. In the present era, the above drugs are rarely used in the United States and this should be kept in mind when choosing an antiarrhythmic therapy.

Management has the twin goals of stabilizing the patient while preventing spread among caregivers. Arrhythmias in COVID-19 patients should generally be managed as indicated in a patient with an acute infectious process. Whenever possible, the underlying cause should be treated first. Aerosol-generating procedures should be minimized whenever possible. Bradyarrhythmias are usually transient and, when necessary, temporary pacing with an active fixation lead should be considered as an initial option before placing a permanent pacemaker. When the implantation of a permanent pacemaker is necessary, however, it should be kept in mind that electrocautery is an aerosol-generating procedure, requiring appropriate precautions.

Treatment of atrial fibrillation and flutter should be conservative and directed at achieving rate control rather than rhythm control, unless there is hemodynamic compromise or heart failure. This is recommended so as to avoid drug–drug interactions and in recognition of the fact that the atrial arrhythmias are a consequence of the underlying pathology. Calcium channel or β-receptor blockers should be the agents of choice, supplemented by digoxin when necessary. Anticoagulation is of special importance; COVID-19 infection is associated with a hypercoagulable state and the CHA_2_DS_2_-VASc score may underestimate the stroke risk.^12^ A retrospective study suggested that pulmonary embolism occurs in 24% of hospitalized COVID-19 patients and in up to 50% of those requiring intensive care.^13^ This is an additional reason for prophylactic anticoagulation. Pulmonary embolism may also precipitate atrial fibrillation. Anticoagulation should be initiated early to avoid the need for transesophageal echocardiography, an aerosol-generating procedure. Heparin may be the anticoagulant of choice in inpatients due to its anti-inflammatory and antiviral properties.^14^ When cardioversion is necessary, cardiac computed tomography may be used to avoid transesophageal echocardiography.

Rhythm control may become necessary in the face of hemodynamic compromise. Electrical or chemical cardioversion can be performed in such cases, but recurrence is likely in severely ill patients. Antiarrhythmics may be used to prevent recurrence when this is rendered imperative; the choice of drug depends upon cardiac involvement by COVID-19, drug–drug interactions, and potential compromise of the route of elimination of the drug. In critically ill patients with atrial arrhythmias that cause hemodynamic compromise, amiodarone is the drug of choice to prevent recurrence. Physicians may be justifiably reluctant to use amiodarone in patients with COVID-19 pneumonia because of potential lung toxicity, but it should be remembered that, in patients without lung compromise, this effect takes months or years to become clinically apparent. However, whether this is also true in COVID-19 pneumonia patients is unknown and intravenous procainamide, where available, may be an acceptable substitute. Some earlier reviews, when hydroxychloroquine/azithromycin and fingolimod were commonly used, recommended the use of propafenone. These drugs are no longer used in the United States due to toxicity and lack of efficacy. It is also now evident that cardiac involvement is quite prevalent, especially in sicker patients. We do not recommend the use of class 1C agents in patients with cardiac involvement due to the risk of proarrhythmia.

Ventricular tachycardia should be treated in COVID-19 patients as it is in non–COVID-19 patients in general, recognizing that ventricular fibrillation is a common final event in moribund patients. The importance of treating underlying causes (eg, hypoxia, electrolyte imbalance) is emphasized. In patients with myocardial ischemia, lidocaine and esmolol are recommended for initial treatment. Amiodarone is appropriate, alone or in combination with lidocaine for ventricular tachycardia storm. It is important to distinguish polymorphic ventricular tachycardia, usually due to ischemia, from torsades de pointes. The latter is treated as it is in non–COVID-19 patients by discontinuing offending drugs, ensuring electrolyte homeostasis, and enacting temporary pacing at a faster rate. A wearable cardioverter-defibrillator (LifeVest; Zoll Corporation, Chelmsford, MA, USA) should be considered for high-risk patients as a bridge to implantable cardioverter-defibrillator placement after recovery.

The impact of COVID-19 on patients with inherited arrhythmias and ion channel abnormalities deserves special consideration. The electrolyte abnormalities associated with COVID-19, especially hypokalemia and hypomagnesemia, need to be detected and prevented in patients with various long QT syndromes, while the continuation of β-blockers is important due to the hyperadrenergic state that occurs during severe illness. Fever can provoke ventricular tachycardia in patients with Brugada syndrome and type 2 long QT syndrome and should be vigorously managed with acetaminophen and nonsteroidal anti-inflammatory drugs. Patients with type 1 Brugada syndrome need inpatient monitoring while febrile; the wearable cardioverter-defibrillator may be appropriate to prescribe when there is a shortage of beds. In patients with catecholaminergic polymorphic ventricular tachycardia, β-blockers and flecainide should be continued during illness and intravenous sympathomimetics should be avoided.

## Conclusion

The COVID-19 pandemic is the greatest health care challenge in living memory. We wish to honor the silent sacrifice of millions of health care workers worldwide who have risked their health and that of their families to care for patients. We also applaud the extraordinary achievements of more millions of scientists and researchers the world over, who sequenced the SARS-CoV-2 genome within weeks of its emergence on the global stage and have since developed highly effective monoclonal antibodies and preventive vaccines within months. This feat has no parallel in the history of medicine.

## Figures and Tables

**Table 1:** COVID-19 Drugs and Arrhythmic Effects

Drug	Class	FDA Approval	Arrhythmic Effect	Efficacy
Bamlanivimab	Monoclonal antibody	EUA	None known	+
Casirivimab/imdevimab	Monoclonal antibody	EUA	None known	+
Remdesivir	Antiviral	EUA	None known	+
Dexamethasone	Glucocorticoid	Yes	None known	+
Baricitinib	JAK inhibitor	EUA	None known	+
Chloroquine/hydroxychloroquine	Antimalarial	Yes (off label)	QT prolongation	−
Azithromycin	Antibiotic	Yes (off label)	QT prolongation	−
Lopinavir/ritonavir	Antiviral	Yes (off label)	Potentiation of antiarrhythmics	−
Fingolimod	Sp1 receptor modulator	Yes (off label)	Heart block, QT prolongation	−
Tocilzumab	IL-6 receptor blocker	Yes (off label)	None known	−

EUA: emergency use approval; FDA: United States Food and Drug Administration; IL-6: interleukin-6; JAK: Janus kinase.
